# Schisandrae Fructus Supplementation Ameliorates Sciatic Neurectomy-Induced Muscle Atrophy in Mice

**DOI:** 10.1155/2015/872428

**Published:** 2015-05-12

**Authors:** Joo Wan Kim, Sae-Kwang Ku, Ki Young Kim, Sung Goo Kim, Min Ho Han, Gi-Young Kim, Hye Jin Hwang, Byung Woo Kim, Cheol Min Kim, Yung Hyun Choi

**Affiliations:** ^1^Research Institute, Bio-Port Korea INC, Marine Bio-Industry Development Center, Busan 619-912, Republic of Korea; ^2^Department of Anatomy and Histology, College of Korean Medicine, Daegu Haany University, Gyeongsan 712-715, Republic of Korea; ^3^Department of Biochemistry, Dong-eui University College of Korean Medicine, Busan 614-052, Republic of Korea; ^4^Laboratory of Immunobiology, Department of Marine Life Sciences, Jeju National University, Jeju 690-756, Republic of Korea; ^5^Anti-Aging Research Center and Blue-Bio Industry RIC, College of Natural Sciences & Human Ecology, Dong-eui University, Busan 614-714, Republic of Korea; ^6^Department of Food and Nutrition, College of Natural Sciences & Human Ecology, Dong-eui University, Busan 614-714, Republic of Korea; ^7^Department of Life Science and Biotechnology, College of Natural Sciences & Human Ecology, Dong-eui University, Busan 614-714, Republic of Korea; ^8^Department of Biochemistry, Busan National University College of Medicine, Yangsan 626-870, Republic of Korea

## Abstract

The objective of this study was to assess the possible beneficial skeletal muscle preserving effects of ethanol extract of Schisandrae Fructus (EESF) on sciatic neurectomy- (NTX-) induced hindlimb muscle atrophy in mice. Here, calf muscle atrophy was induced by unilateral right sciatic NTX. In order to investigate whether administration of EESF prevents or improves sciatic NTX-induced muscle atrophy, EESF was administered orally. Our results indicated that EESF dose-dependently diminished the decreases in markers of muscle mass and activity levels, and the increases in markers of muscle damage and fibrosis, inflammatory cell infiltration, cytokines, and apoptotic events in the gastrocnemius muscle bundles are induced by NTX. Additionally, destruction of gastrocnemius antioxidant defense systems after NTX was dose-dependently protected by treatment with EESF. EESF also upregulated muscle-specific mRNAs involved in muscle protein synthesis but downregulated those involved in protein degradation. The overall effects of 500 mg/kg EESF were similar to those of 50 mg/kg oxymetholone, but it showed more favorable antioxidant effects. The present results suggested that EESF exerts a favorable ameliorating effect on muscle atrophy induced by NTX, through anti-inflammatory and antioxidant effects related to muscle fiber protective effects and *via* an increase in protein synthesis and a decrease in protein degradation.

## 1. Introduction

Muscle atrophy, known as a sarcopenia, is defined as a loss of muscle mass resulting from a reduction in muscle fiber area or density due to a decrease in muscle protein synthesis and an increase in protein breakdown [1, 2]. Many conditions are associated with muscle atrophy, such as aging, denervation, disuse, starvation, severe injury and inflammation, prolonged bed rest, glucocorticoid treatment, sepsis, cancer, and other cachectic diseases [3–5]. Moreover, the loss of skeletal muscle mass has a profound effect on a patient's daily life, especially physical activity. The resulting reduction in physical activity induces further skeletal muscle atrophy, leading to a vicious circle of the atrophic process. The disuse of the muscles will also lead to disuse atrophy, while neurogenic atrophy is muscle atrophy that results from damage to the nerve that stimulates the muscle [4, 6, 7].

There is a common genetic program involved in muscle proteolysis regardless of its etiology; however the signaling pathways that modulate this system are distinct [6, 8]. It is well established that the serum levels of proinflammatory cytokines, particularly interleukin- (IL-) 1 and tumor necrosis factor- (TNF-) *α*, are elevated in cachectic patients contributing to muscle atrophy [8, 9]. Moreover, the oxidative stress is an important inducer of muscle atrophy in both disuse and muscle cachexia [10]. In addition, apoptosis of muscle fibers is also involved in the early phase of muscle atrophy regardless of its etiology [11, 12]. Up till now, various animal models of skeletal muscle atrophy have been used in research fields including those of denervation [13], unloading [14, 15], immobilization [16], starvation [17], and administration of dexamethasone [18]. Among them, denervation (sciatic neurectomy; NTX) causes numerous changes in contractile, electrical, metabolic, and molecular properties of the muscle fiber membrane and sarcoplasm in hindlimb skeletal muscles [19, 20]. Previous studies have shown that denervation alters interstitial spaces between muscle fibers: mononucleated cells are infiltrated into interstitial spaces of muscle fibers, and fibrosis occasionally occurs in the muscle [21–24]. These findings suggest that NTX-induced hindlimb muscle atrophy is a useful and rapid model* in vivo* for screening the agents that can reverse the abnormalities in muscle atrophy.

Schisandrae Fructus, the dried fruits of* Schisandra chinensis* (Turcz.) Baill. (Magnoliaceae), one of the most famous herbal medicines, has been extensively used in Asia including Korea, China, and Japan with Russia [25, 26]. It was originally used as a tonic and has been traditionally used for the treatment of many uncomfortable symptoms, such as cough, dyspnea, dysentery, insomnia, and amnesia for a long time [25, 26]. Previous reports have shown that Schisandrae Fructusand its related compounds possess various biological activities such as antioxidant, anti-inflammatory, antimicrobial, antiseptic, antiaging, hepatoprotective, immunostimulating, and anticancer effects [27–31]. In addition, recent reports have suggested that Schisandrae Fructus has favorable effects on diabetes and it is related [32–34] with smooth muscle relaxant effects [35, 36]. However, the effect of Schisandrae Fructus on muscle atrophy has not yet been evaluated. In the present study, we assessed the possible beneficial skeletal muscle preserving effects of ethanol extract of Schisandrae Fructus (EESF) on sciatic NTX-induced hindlimb muscle atrophy in mice. In this experiment, calf muscle atrophy was induced by unilateral right sciatic NTX according to previous studies [23, 37]. In order to investigate whether administration of EESF prevents or improves sciatic NTX-induced muscle atrophy with possible action mechanisms, 125, 250, and 500 mg/kg of EESF were administered orally, once a day for 28 days from 2 weeks after NTX in mice for the therapeutic study.

## 2. Materials and Methods

### 2.1. Test Substance

Schisandrae Fructus were collected around Mungyeong- (Gyeongsangbuk-do, Republic of Korea) and washed three times with tap water before storage at −20°C. The frozen samples were lyophilized and homogenized using a grinder before extraction. The materials were extracted with 20% ethanol (EESF) at room temperature for 24 h, filtered, and concentrated using a rotary vacuum evaporator (Buchi Rotavapor R-144, BÜCHI Labortechnik, Flawil, Switzerland). The extract was dissolved in dimethyl sulfoxide (DMSO; Sigma-Aldrich) as a 50 mg/mL stock solution and stored at 4°C until use. In addition, oxymetholone, 17*β*-hydroxy-2-hydroxymethylidene-17*α*-methyl-3-androstanone (Celltrion Pharm Inc., Jincheon, Republic of Korea), which is an orally active 17*α*-alkylated anabolic-androgenic steroid first described by Ringold et al. [38], was used as the reference drug; it was also stored in a refrigerator at 4°C to protect from light and degeneration, until use.

### 2.2. Animals and Experimental Design

Total 60 adult SPF/VAF Outbred CrljOri:CD1 (ICR) mice (Orient Bio Inc., Seongnam, Republic of Korea) weighing 29~32 g were used in this experiment after allowing for 7 days of acclimatization. The animals were allocated five per polycarbonate cage in a temperature (20–25°C) and humidity (40–45%) controlled room. The light : dark cycle was 12 hr : 12 hr and normal rodent pellet diet (Samyang Co., Seoul, Republic of Korea) and water were supplied during acclimatization* ad libitum*. After acclimatization, unilateral sciatic NTX was performed to induce hindlimb muscle atrophy according to the previously established method [23, 37]. Briefly, all of the mice were anesthetized with 2 to 3% isoflurane (Hana Pharm. Co., Hwasung, Republic of Korea) in the mixture of 70% N_2_O and 28.5% O_2_, and anesthesia was maintained with 1 to 1.5% isoflurane in the mixture of 70% N_2_O and 28.5% O_2_. Body temperature was maintained at 37.0 ± 1°C, and mice were subjected to operation. Right sciatic neurectomy was performed by removing a 0.5 cm section of the nerve in the mid thigh, and a sham operation (nerve identification) was performed in the case of the sham groups as follows: animals were randomly divided into 6 groups of 10 mice each at 2 weeks after NTX treatment; sham control, NTX control, oxymetholone-treated group, and EESF-treated groups (125, 250, and 500 mg/kg) based on the body weights (37.61 ± 1.63 g, range 35.70~40.40 g in the vehicle sham control group; 37.24 ± 2.20 g, range 34.00~43.90 g in the NTX group) and calf thicknesses (2.86 ± 0.11 mm, range 2.71~3.00 mm in the vehicle sham control group; 2.30 ± 0.06 mm, range 2.18~2.41 mm in the NTX group). The dosage of oxymetholone was selected as 50 mg/kg based on the previous efficacy test in mice [39, 40]. Prepared and stored EESF stock solution (500 mg/mL in DMSO) was 10-, 20-, or 40-fold diluted as 50, 25, and 12.5 mg/mL by distilled water and then orally administered in a volume of 10 mL/kg of body weight as matched as 500, 250, and 125 mg/kg of body weight, by oral gavages, once a day for 28 days from 2 weeks after NTX, respectively. In addition, oxymetholone was also dissolve in distilled water as 5 mg/mL concentrations and orally administered in a volume of 10 mL/kg of body weight as matched as 50 mg/kg of body weight as same period as EESF. An equal volume of distilled water was administered orally in sham and NTX control mice, instead of EESF or oxymetholone. This experiment was conducted according to the international regulations for the use and welfare of laboratory animals and was approved by the Institutional Animal Care and Use Committee of Daegu Haany University (Gyeongsan, Republic of Korea) (Approval number DHU2013-077).

### 2.3. Body Weight Measurements

Body weights of each mouse were measured from the day of NTX to sacrifice using an automatic electronic balance (Precisa Instrument, Dietikon, Switzerland). In addition, the body weight gains during the four-week period of test substance administration were also calculated to reduce individual differences as follows:

 Body weight (g) gains during the total 4 week period of administration = body weight at sacrifice – body weight at the first administration (2 weeks after NTX).

### 2.4. Calf and Gastrocnemius Muscle Thickness Measurements

The thickness of NTX-operated hind calf muscles (right side) was measured at the time of NTX, 1 day before the start of administration, first administration, 1, 7, 14, 21, and 27 days, and at sacrifice using an electronic digital caliper (Mitutoyo, Tokyo, Japan) as mm/mouse levels. In addition, the gastrocnemius muscle thicknesses of both sides of the hindlimb were also measured after muscle exposure at sacrifice to reduce the differences due to surrounding tissues using the same methods, and the changes in calf thickness during the four week period of test substance administration were also calculated to reduce individual differences as follows:

 Calf thickness changes (mm) after 4 weeks of continuous administration = calf thickness at sacrifice – calf thickness at the first administration (2 weeks after NTX).

### 2.5. Gastrocnemius Muscle Weight Measurements

After performing gastrocnemius muscle thickness measurement at sacrifice, the gastrocnemius muscle masses were carefully separated from the tibia and fibula bones. Then, weights of individual induced gastrocnemius muscle masses were measured at g levels (absolute wet-weights) using an automatic electronic balance and, to reduce the differences due to individual body weights, relative weights (% of body weights) were also calculated using body weight at sacrifice and absolute weight as follows:

 Relative muscle mass weights (% of body weights) = ((absolute organ weights/body weight at sacrifice) × 100).

### 2.6. Calf Muscle Strength Measurements

At 1 hr after the last 28th administration of vehicle, oxymetholone, or SF, calf muscle strengths of individual mice were measured as tensile strengths using a computerized testing machine (SV-H1000, Japan Instrumentation System Co., Tokyo, Japan) in Newton (N). Briefly, animals were restrained in machines using two separate 1-0 silk suture ties on the NTX-operated ankle and chest, and the peak tensile loads were recorded as calf muscle strengths, when knee angles reached 0° (10~20 mm distances) in this experiment.

### 2.7. Serum Biochemistry

To obtain the sera for blood biochemistry, blood samples in a separation tube were centrifuged at 3,000 rpm for 10 min on the day of necropsy. Serum creatine and creatine kinase (CK) levels were measured using an autoanalyzer (Hemagen Analyst, Hemagen Diagnostic Inc., Columbia, MD, USA), and lactate dehydrogenase (LDH) levels were measured using another automated serum biochemistry analyzer (Spotchem SP-4410, Arkray Inc., Tokyo, Japan), respectively.

### 2.8. Antioxidant Defense Systems

After performing measurements of muscle mass weights, gastrocnemius muscles were separated, and the malondialdehyde (MDA), reactive oxygen species (ROS), and glutathione (GSH) contents, catalase (CAT), and superoxide dismutase (SOD) enzyme activities in individual muscles were assessed.

#### 2.8.1. Muscle Homogenate Preparation

Separated gastrocnemius muscles were weighed and homogenized in ice-cold 0.01 M Tris-HCl (pH 7.4) and then centrifuged at 12,000 g for 15 min as described by Del Rio et al. [41]. Contents of total protein were measured by the previously described method [42] using bovine serum albumin (BSA, Invitrogen, Carlsbad, CA, USA) as the internal standard.

#### 2.8.2. Determination of Lipid Peroxidation

The levels of muscle lipid peroxidation were determined by estimating MDA with the thiobarbituric acid test at 525 nm absorbance using UV/Vis spectrometer (OPTIZEN POP, Mecasys Co., Daejeon, Republic of Korea) as nM of MDA/g tissue [43].

#### 2.8.3. Determination of Reactive Oxygen Species

Skeletal muscles were homogenized, and ROS level analysis was performed using 2′,7′-dichlorofluorescein diacetate fluorescent dye as a probe and fluorescence density was measured at 490/520 nm according to manufactory guidance (ROS assay kit; Abcam, Cambridge, MN, USA) and the measured values of optical density (a relative fluorescence unit (RFU)) were corrected by the protein concentrations of samples and were expressed as RFU/*μ*g protein [44].

#### 2.8.4. GSH Content Measurement

Prepared homogenates were mixed with 0.1 mL of 25% trichloroacetic acid (Merck, San Francisco, CA, USA) and then centrifuged at 4,200 rpm for 40 min at 4°C. GSH content was measured at 412 nm absorbance using 2-nitrobenzoic acid (Sigma-Aldrich) as mg/g tissue [45].

#### 2.8.5. Tissue CAT Activity

Decomposition of H_2_O_2_ in the presence of CAT was followed at 240 nm [46]. CAT activity was defined as the amount of enzyme required to decompose 1 nM of H_2_O_2_ per minute, at 25°C and pH 7.8. Results were expressed as U/mg protein.

#### 2.8.6. Tissue SOD Activity

Measurements of SOD activities were made according to Sun et al. [47]. SOD estimation was based on the generation of superoxide radicals produced by xanthine and xanthine oxidase, which react with nitrotetrazolium blue to form formazan dye. SOD activity was then measured at 560 nm by the degree of inhibition of this reaction and was expressed as U/mg protein. One unit of SOD enzymatic activity is equal to the amount of enzyme that diminishes the initial absorbance of nitroblue tetrazolium by 50% during 1 min.

### 2.9. Cytokine Content Measurements

Levels of IL-1*β* and TNF-*α* were measured by sandwich enzyme-linked immunoassay (ELISA) according to the manufacturer's instructions (R&D Systems, Inc., Minneapolis, MN, USA). Approximately 10–15 mg of gastrocnemius muscle samples were homogenized in a tissue grinder containing 1 mL of lysis buffer (PBS containing 2 mM PMSF and 1 mg/mL of aprotinin, leupeptin, and pepstatin A) as described by Clark et al. [48]. A 96-well plate was coated with 2 *μ*g/mL of monoclonal anti-mouse IL-1*β* or TNF-*α* antibody at 4°C overnight and then blocked with 1% BSA in PBS for 1 hr. The plates were washed three times with PBS containing 0.2% Tween 20. Aliquots of tissue lysates were diluted to 100 *μ*L with Hanks' balanced salt solution with calcium and magnesium, 10 mM 4-(2-hydroxyethyl)-1-piperazineethanesulfonic acid, and 1% fetal bovine serum, added to the plates, and then incubated for 2 hrs at room temperature. The plates were washed three times with PBS, and 100 *μ*L aliquots of 0.1 *μ*g/mL biotinylated mouse IL-1*β* or TNF-*α* affinity-purified polyclonal antibody was added and incubated for 2 hrs. After further three washes with PBS containing 0.2% Tween 20, the immune complexes were colorimetrically detected using horseradish peroxidase- (HRP-) streptavidin conjugate. The reaction was halted by the addition of 1 M H_2_SO_4_, and the absorbance at 450 nm was measured using a microplate reader (Tecan; Männedorf, Switzerland).

### 2.10. Quantitative RT-PCR

RNA was extracted using Trizol reagent (Invitrogen, Carlsbad, CA, USA), according to the method recommended. The RNA concentrations and quality were determined by CFX96 Real-Time System (Bio-Rad, Hercules, CA, USA). To remove contaminating DNA, samples were treated with recombinant DNase I (DNA-free; Ambion, Austin, TX, USA). RNA was reverse transcribed using the reagent High-Capacity cDNA Reverse Transcription Kit (Applied Biosystems, Foster City, CA, USA) according to the manufacturer's instructions. The internal control was 18s ribosomal RNA. The sequences of the PCR oligonucleotide primers are listed in Table 1.

### 2.11. Histopathology

Samples from gastrocnemius muscles were separated and fixed in 10% neutral buffered formalin, then embedded in paraffin, sectioned (3~4 *μ*m), and stained with Hematoxylin and eosin (H&E) for general histopathology [23] or Sirius red for collagen fiber [49], and after that the histopathological profiles of each sample were observed under light microscope (Nikon, Japan). To observe more detailed changes, mean muscle fiber diameter (*μ*m/fiber), number of inflammatory cells infiltrated into the muscle bundles (cells/mm^2^ of muscle bundles), and regions occupied by collagen fibers (%/mm^2^ of muscle bundles) were calculated for general histomorphometrical analysis of gastrocnemius muscle samples using an automated image analyzer (*i*Solution FL ver. 9.1, IMT* i*Solution Inc., Quebec, Canada), according to the previously described methods [15, 23] with some modifications.

### 2.12. Immunohistochemistry

After deparaffinization of prepared gastrocnemius muscle histological paraffin sections, citrate buffer antigen (epitope) retrieval pretreatment was conducted as described previously [50]. Briefly, water bath was preheated with staining dish containing 10 mM citrate buffer (pH 6.0) until the temperature reached 95–100°C. Slides were immersed in the staining dish and the lid was placed loosely on the staining dish. Slides were incubated for 20 minutes and the water bath was turned off. The staining dish was kept at room temperature and the slides were allowed to cool for 20 minutes. After epitope retrieval, sections were immunostained using avidin-biotin complex (ABC) methods for caspase-3, poly (ADP-ribose) polymerase (PARP), nitrotyrosine, 4-hydroxynonenal (4-HNE), inducible nitric oxide synthase (iNOS), cyclooxygenase-2 (COX-2), TNF-*α*, and myostatin (Table 2) according to the previous study [51]. Briefly, endogenous peroxidase activity was blocked by incubation in methanol and 0.3% H_2_O_2_ for 30 minutes, and nonspecific binding of immunoglobulin was blocked with normal horse serum blocking solution (Vector Lab., Burlingame, CA, USA. Dilution 1 : 100) for 1 hr in the humidity chamber. Sections were treated with primary antiserum (Table 2) overnight at 4°C in the humidity chamber and then incubated with biotinylated universal secondary antibody (Vector Lab., Dilution 1 : 50) and ABC reagents (Vectastain Elite ABC Kit, Vector Lab., Dilution 1 : 50) for 1 hr at room temperature in the humidity chamber and, finally, reacted with peroxidase substrate kit (Vector Lab.) for 3 min at room temperature. All sections were rinsed PBS for 3 times, between steps. The cells or muscle fibers occupying more than 20% of immunoreactivities, the density, of each antiserum for caspase-3, PARP, nitrotyrosine, 4-HNE, iNOS, COX-2, TNF-*α*, and myostatin as compared with intact muscles were regarded as positive, and the mean number of caspase-3, PARP, nitrotyrosine, 4-HNE, iNOS, and myostatin immunoreactive fibers or COX-2 and TNF-*α* immunoreactive cells dispersed in the mm^2^ of muscle bundles was counted using an automated image analysis process as per the established methods [52, 53] with some of our modifications, respectively. The histopathologist was blinded to the group distribution while performing the analysis.

### 2.13. Statistical Analyses

Multiple comparison tests were conducted for different dose groups. Variance homogeneity was examined using Levene's test [54]. When Levene's test showed no significant deviations from variance homogeneity, the obtained data were analyzed by one-way ANOVA test followed by least-significant difference multicomparison (LSD) test to determine which pairs of group comparison were significantly different. When significant deviations from variance homogeneity were observed in Levene's test, a nonparametric comparison test, Kruskal-Wallis *H* test was conducted. When a significant difference was observed in the Kruskal-Wallis *H* test, the Mann-Whitney *U* (MW) test was conducted to determine the specific pairs of group comparison, which were significantly different. Statistical analyses were conducted using SPSS for Windows (Release 14 K, SPSS Inc., USA) [55]. Differences were considered significant at *p* < 0.05.

## 3. Results

### 3.1. Effects of EESF Supplementation on Body Weight Changes

**Figure 1:**
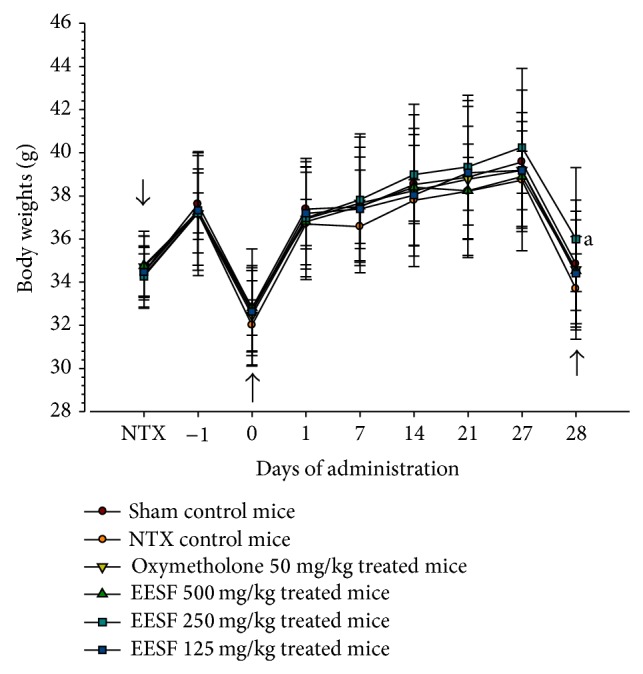
Effects of EESF supplementation on body weight changes in mice with NTX-induced muscle atrophy. Body weights of each mouse were measured from the day of NTX to sacrifice as described in Section 2. Values are expressed as mean ± S.D. of 10 mice (EESF, Schisandrae Fructus ethanol extract; NTX, Sciatic neurectomy). Day −1 means 1 day before the start of administration, at 13 days after NTX surgery. Zero means at the start of administration, at 14 days after NTX. Twenty-eight means 28 days after the start of administration, at sacrifice. All animals were fasted overnight before NTX, first administration, and sacrifice (arrows). ^a^ *p* < 0.05 as compared with NTX control mice by the LSD test.

Except for the significant (*p* < 0.05) increases in body weights detected in 250 mg/kg EESF-treated mice limited to the day of sacrifice as compared with NTX vehicle control mice, no sciatic NTX or test substance treatment-related meaningful or significant changes in the body weights were demonstrated during 6 weeks of the experimental period in this experiment (Figure 1). In addition, there were no critical changes in body weight gains during the 4-week test substance administration period in all oxymetholone-treated mice or EESF-treated mice as compared with sham or NTX control mice, except for the significant (*p* < 0.01 or *p* < 0.05) increases in body weight gains detected in 250 mg/kg EESF-treated mice as compared to those in sham and NTX control mice, respectively (Table 3).

### 3.2. EESF Supplementation Mitigated NTX-Induced Loss of Calf Thickness

**Figure 2:**
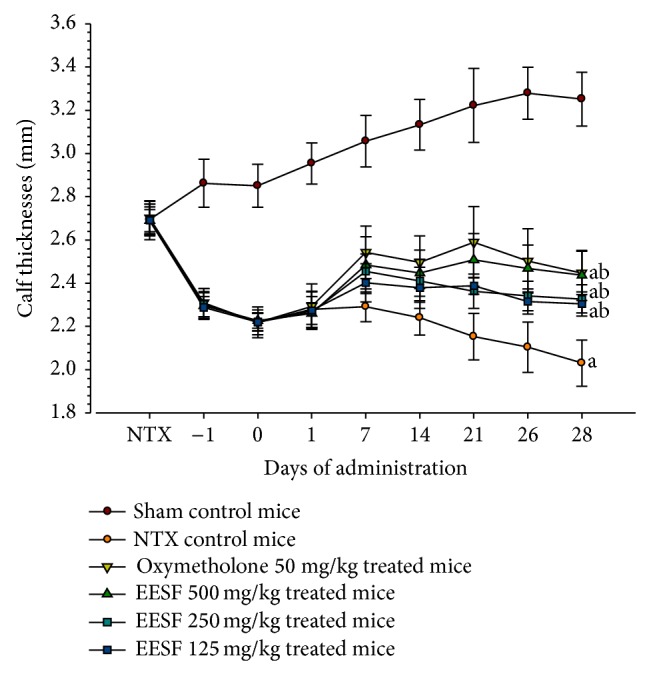
Effects of EESF supplementation on calf thickness changes in mice with NTX-induced muscle atrophy. The thickness of NTX operated hind calf (right side) was measured at NTX, 1 day before the start of administration, first administration, 1, 7, 14, 21, and 27 days, and sacrifice. Values are expressed as mean ± S.D. of 10 mice. Day −1 means 1 day before the start of administration, at 13 days after NTX surgery. Zero means at the start of administration and at 14 days after NTX. Twenty-eight means 28 days after the start of administration, at sacrifice. ^a^ *p* < 0.01 as compared with sham control mice by the LSD test. ^b^ *p* < 0.01 as compared with NTX control mice by the LSD test.

Significant (*p* < 0.01) decreases in calf thicknesses were demonstrated in NTX control mice as compared with sham control mice from 1 day before first administration of the test substance to throughout the experimental period (Figure 2). Accordingly, the calf thickness after 4 weeks was significantly (*p* < 0.01) decreased in NTX control mice as compared with sham vehicle control mice (Table 4). However, these decreases in calf thicknesses were significantly (*p* < 0.01) diminished by treatment with oxymetholone and EESF from 7 days after first administration of the drug, and the calf thickness was restored during the drug administration period (*p* < 0.01) in these groups as compared with NTX control group, respectively (Table 4).

### 3.3. EESF Supplementation Ameliorated NTX-Induced Loss of Gastrocnemius Muscle Thickness

**Figure 3:**
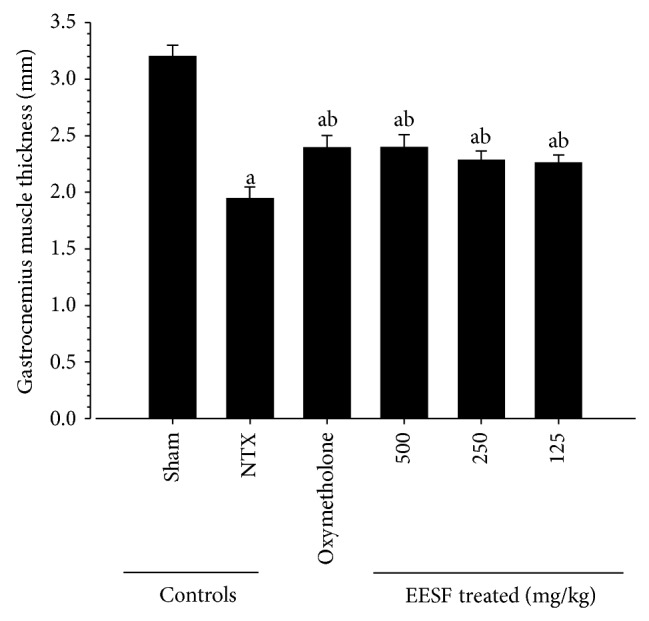
Effects of EESF supplementation on changes in gastrocnemius muscle thicknesses after muscle exposure in mice with NTX-induced muscle atrophy mice. The gastrocnemius muscle thicknesses of both sides of the hindlimb were measured after muscle exposure at sacrifice. Values are expressed as mean ± S.D. of 10 mice. ^a^ *p* < 0.01 as compared with sham control mice by the LSD test. ^b^ *p* < 0.01 as compared with NTX control mice by the LSD test.

Significant (*p* < 0.01) decreases in gastrocnemius muscle thicknesses after muscle exposure (through skin removal) were observed in NTX control mice as compared with sham vehicle control mice (Figure 3). However, significant (*p* < 0.01) increases in gastrocnemius muscle thicknesses were detected in oxymetholone-treated mice and EESF-treated mice as compared with NTX control mice, respectively.

### 3.4. EESF Supplementation Ameliorated NTX-Induced Loss of Gastrocnemius Muscle Mass Weights

**Figure 4:**
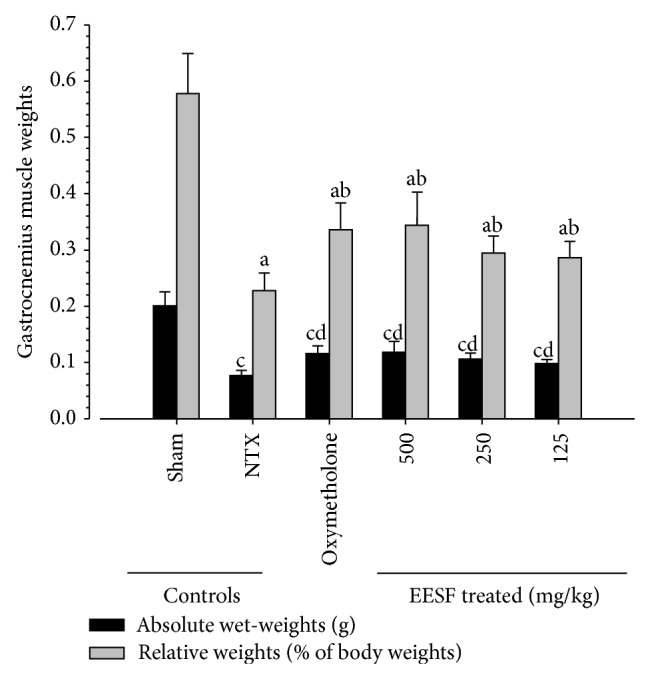
Effects of EESF supplementation on changes in gastrocnemius muscle weights in mice with NTX-induced muscle atrophy. After performing gastrocnemius muscle thickness measurement at sacrifice, the gastrocnemius muscle masses were carefully separated from the tibia and fibula bones. Then, weights of individual induced gastrocnemius muscle masses were measured at g levels (absolute wet-weights), and relative weights (% of body weights) were also calculated. Values are expressed as mean ± S.D. of 10 mice. ^a^ *p* < 0.01 as compared with sham control mice by the LSD test. ^b^ *p* < 0.01 as compared with NTX control mice by the LSD test. ^c^ *p* < 0.01 as compared with sham control mice by the MW test. ^d^ *p* < 0.01 as compared with NTX control mice by the MW test.

Significant (*p* < 0.01) decreases in absolute wet-weight and relative weights of gastrocnemius muscle mass were demonstrated in NTX control mice as compared with sham vehicle control mice (Figure 4). However, significant (*p* < 0.01) increases in gastrocnemius muscle mass were observed in oxymetholone-treated mice and EESF-treated mice as compared with NTX control mice, respectively.

### 3.5. EESF Supplementation Attenuated NTX-Induced Loss of Calf Muscle Strengths

**Figure 5:**
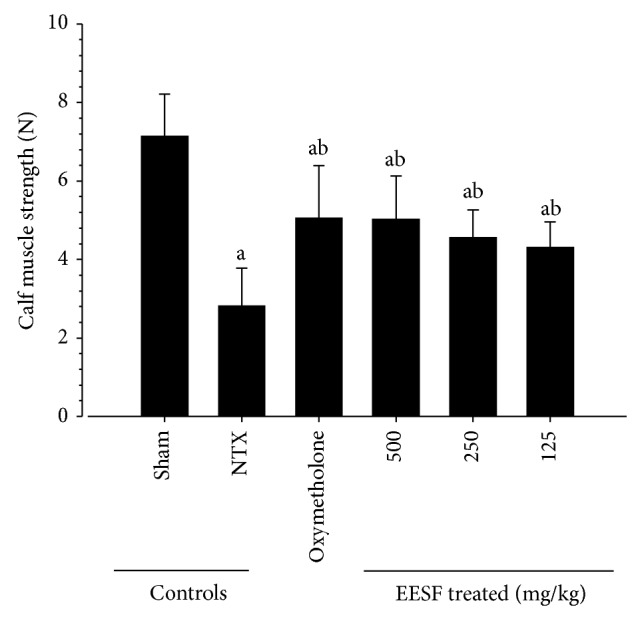
Effects of EESF supplementation on changes in calf muscle strengths in mice with NTX-induced muscle atrophy mice. At 1 hr after the last 28th administration of vehicle, oxymetholone, or EESF, calf muscle strengths of individual mice were measured as tensile strengths using a computerized testing machine in Newton (N). Values are expressed as mean ± S.D. of 10 mice (N = Newton). ^a^ *p* < 0.01 as compared with sham control mice by the LSD test. ^b^ *p* < 0.01 as compared with NTX control mice by the LSD test.

Significant (*p* < 0.01) decreases in tensile strengths of calf muscles were demonstrated in NTX control mice as compared with sham vehicle control mice (Figure 5). However, significant (*p* < 0.01) increases in calf muscle strengths were observed in oxymetholone-treated mice and EESF-treated mice as compared with NTX control mice, respectively. In particular, EESF-treated mice showed dose-dependent increases in calf muscle strengths as compared with NTX control mice.

### 3.6. Effects of EESF Supplementation on Serum Biochemistry

Significant (*p* < 0.01) decreases in serum creatine levels and increases in serum CK and LDH levels were demonstrated in NTX control mice as compared with sham vehicle control mice. However, significant (*p* < 0.01) increases in serum creatine levels were observed in oxymetholone-treated mice and EESF-treated mice as compared with NTX control mice, along with significant (*p* < 0.01 or *p* < 0.05) decreases in serum CK and LDH levels, respectively (Table 5).

### 3.7. Effects of EESF Supplementation on the Gastrocnemius Muscle Antioxidant Defense Systems

Significant (*p* < 0.01) increases in muscle lipid peroxidation, elevation of MDA levels, and ROS content were observed in NTX control mice as compared with sham control mice (Table 6). However, the elevated levels were significantly (*p* < 0.01) reduced by treatment with EESF in a dose-dependent manner. In addition, the increases in lipid peroxidation and ROS content were also significantly (*p* < 0.01) decreased in oxymetholone-treated mice as compared with NTX control mice. In addition, a significant (*p* < 0.01) decrease in the muscle endogenous antioxidant, GSH content, and antioxidative enzyme (SOD and CAT) activity was detected in NTX control mice as compared with sham control mice; however, these decreases were significantly (*p* < 0.01) dose-dependently diminished by continuous oral treatment with oxymetholone and EESF for 28 days, respectively.

### 3.8. Effects of EESF Supplementation on the Gastrocnemius Muscle Cytokine Content

Significant (*p* < 0.01) increases in the levels of gastrocnemius muscle proinflammatory cytokines such as IL-1*β* and TNF-*α* were observed in NTX control mice as compared with sham control mice (Table 7). However, these elevations were significantly (*p* < 0.01) decreased by treatment with EESF in a dose-dependent manner. In addition, the IL-1*β* and TNF-*α* levels in oxymetholone-treated mice were significantly (*p* < 0.01) decreased as compared with those in NTX control mice in this experiment.

### 3.9. Effects of EESF Supplementation on the Gastrocnemius Muscle mRNA Expression Levels

Significantly (*p* < 0.01) increased gastrocnemius muscle mRNAs expression levels of Atrogin-1 and RING-finger protein-1 (MuRF1), which are involved in protein degradation, myostatin, a potent negative regulator of muscle growth, and Sirtuin 1 (SIRT1), a representative inhibitor of muscle regeneration, were observed in NTX control mice as compared with sham control mice (Table 8). However, these elevations were significantly (*p* < 0.01) dose-dependently decreased by treatment with oxymetholone and EESF as compared with those in NTX control mice (Table 8). On the other hand, significant (*p* < 0.01) decreases in mRNA expression levels of phosphatidylinositol 3-kinase (PI3K), Akt1, adenosine A1 receptor (A1R), and transient receptor potential cation channel subfamily V member 4 (TRPV4), which are involved in activating protein synthesis and muscle growth, were observed in NTX control mice as compared with intact control mice. However, significant (*p* < 0.01) increases in their expression levels were demonstrated in EESF-treated mice as compared with NTX control mice, in a concentration-dependent manner.

### 3.10. Effects of EESF Supplementation on the Gastrocnemius Muscle Histopathology

**Figure 6:**
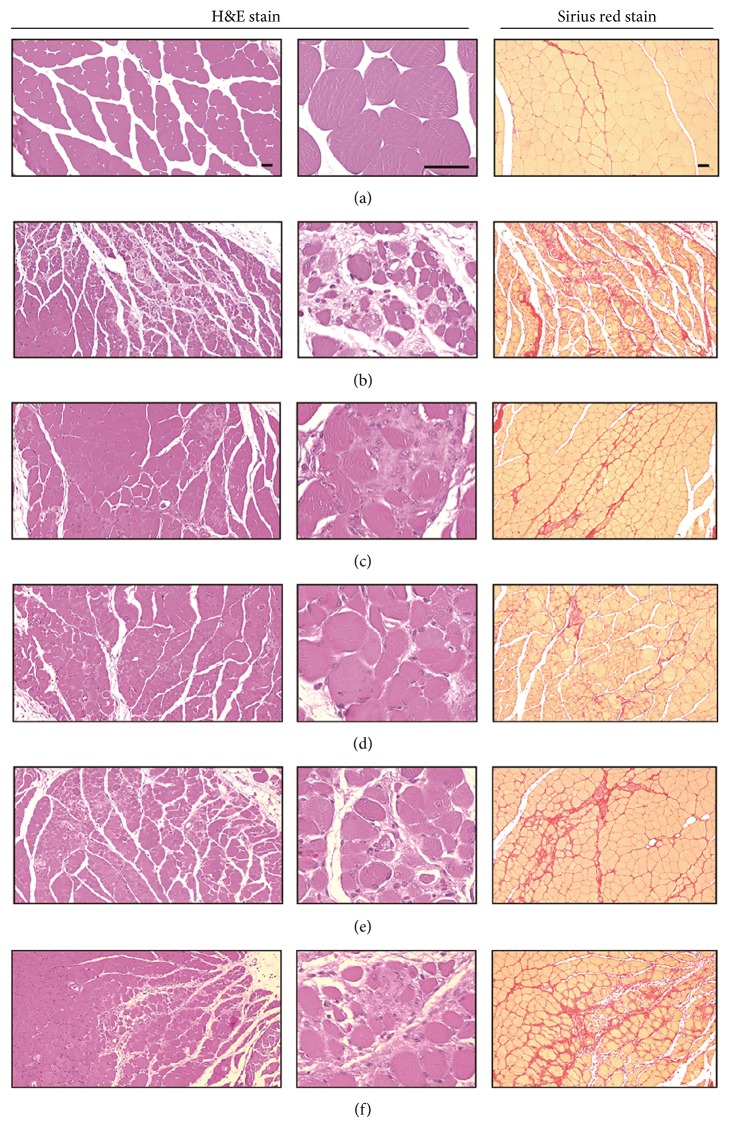
Representative gastrocnemius muscle histology in samples taken from sham mice or mice with NTX-induced muscle atrophy. Samples from gastrocnemius muscles were separated and fixed in 10% neutral buffered formalin, then embedded in paraffin, sectioned, and stained with H&E for general histopathology or Sirius red for collagen fiber, and after that the histopathological profiles of each sample were observed under light microscope ((a) intact vehicle control mice; (b) NTX control mice; (c) 50 mg/kg oxymetholone-treated mice; (d) 500 mg/kg EESF orally treated mice; (e) 250 mg/kg EESF mg/kg orally treated mice; (f) 125 mg/kg EESF orally treated mice; scale bars = 40 *μ*m).

Marked and classic muscle atrophic changes including decline in the number of muscle fibers, inflammatory cell infiltration, and focal fibrosis in muscle bundles were induced by NTX and, accordingly, significant (*p* < 0.01) decreases in the mean muscle fiber diameter, increases in the mean number of inflammatory cells infiltrating into, and percentages of regions occupied by collagen fibers in muscle bundles were detected in NTX control mice as compared with sham control mice (Table 9 and Figure 6). However, these NTX-treatment related gastrocnemius muscle atrophic changes were dramatically and significantly (*p* < 0.01) reduced by treatment with EESF in a dose-dependent manner. In addition, the muscle atrophic changes were also significantly (*p* < 0.01) diminished in oxymetholone-treated mice as compared with NTX control mice in our experiment.

### 3.11. Effects of EESF Supplementation on the Gastrocnemius Muscle Immunohistochemistry

**Figure 7:**
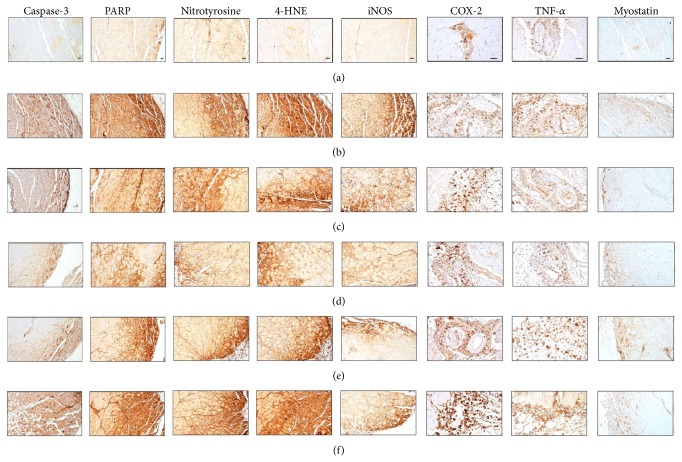
Representative gastrocnemius muscle caspase-3, PARP, nitrotyrosine, 4-HNE, iNOS, COX-2, TNF-*α*, and myostatin immunoreactivities in samples taken from intact mice or mice with NTX-induced muscle atrophy. After deparaffinization of prepared gastrocnemius muscle histological paraffin sections, citrate buffer antigen (epitope) retrieval pretreatment was conducted. Thereafter, the sections were immunostained using ABC methods for caspase-3, PARP, nitrotyrosine, 4-HNE, iNOS, COX-2, TNF-*α*, and myostatin ((a) intact vehicle control mice; (b) NTX control mice; (c) 50 mg/kg oxymetholone-treated mice; (d) 500 mg/kg EESF orally treated mice; (e) 250 mg/kg EESF mg/kg orally treated mice; (f) 125 mg/kg EESF orally treated mice; Scale bars = 40 *μ*m).

Marked and significant (*p* < 0.01) increases in the apoptotic markers, caspase-3, and PARP immunoreactive fibers in the gastrocnemius muscle bundles were observed in NTX control mice; however, EESF dose-dependently and significantly (*p* < 0.01) normalized these NTX-related changes (Table 10 and Figure 7). In addition, oxymetholone also significantly (*p* < 0.01) decreased these changes as compared with those in NTX control mice in our experiment (Table 10 and Figure 6). EESF and oxymetholone also dose-dependently normalized the increases in oxidative stress markers such as nitrotyrosine and iNOS and the lipid peroxidation marker, 4-HNE, in the gastrocnemius muscle bundles of NTX control mice. Moreover, the increase in inflammatory markers including COX-2 and TNF-*α* observed in the gastrocnemius muscle bundles of NTX control mice was significantly attenuated in EESF-treated mice and oxymetholone-treated mice, which was associated with normalization of NTX-induced increase in myostatin immunoreactive fibers.

## 4. Discussion

Sciatic neurectomy, denervation, can induce muscle atrophy characterized by a reduction in the protein content, organelles, cytoplasm, the fiber diameter, the production of muscle strength, and the resistance to fatigue [4, 8, 9]. Denervation also causes numerous changes in contractile, electrical, metabolic, and molecular properties of the muscle fiber membrane and sarcoplasm in hindlimb skeletal muscles [19, 20]. In the present study, we assessed the possible beneficial skeletal muscle preserving effects of EESF on sciatic NTX-induced hindlimb muscle atrophy in mice. Accordingly, decreases in calf thicknesses and muscle strengths and in gastrocnemius muscle thicknesses and weights at sacrifice were induced by sciatic NTX as a result of calf muscle atrophy. However, EESF dose-dependently diminished these decreases, which can be considered as one of the direct evidences that EESF, as did oxymetholone, ameliorated the NTX-induced calf muscle atrophic changes.

Creatine is a nitrogenous organic acid that occurs naturally and helps to supply energy to all cells in the body, primarily muscle, and plasma creatine levels can be used as a valuable serum biochemical marker for assessing the skeletal muscle activity or amounts [56, 57]. LDH is of medical significance because it is found extensively in body tissues, such as blood cells and heart muscle, and CK, an enzyme expressed by various tissues and cell types, catalyses the conversion of creatine, and consumes adenosine triphosphate. Because they are released during tissue damage, they are markers of common injuries and disease, especially muscle damage. Therefore, the plasma activities of CK and LDH have been commonly used as markers of muscle tissue damage [58, 59]. They are also markedly elevated in disuse muscle atrophy in animals [60]. In the present study, marked decreases in serum creatine levels were demonstrated along with NTX-induced decreases in muscle masses and activities, as in a previous study [60], but treatment with EESF dose-dependently and significantly diminished these NTX-induced decreases in the serum creatine level. Significant elevations in serum CK and LDH levels, indicating muscle damage, were also detected in NTX control mice; however, significant and dose-dependent decreases in serum CK and LDH levels were observed in EESF-treated mice, which can be considered as the indirect evidences suggesting that EESF has favorable and potent muscle preserving effects.

Various toxic substances from lipid peroxidation destroy the surrounding tissues [61], and the oxidative stress is also an important inducer of muscle atrophy in both disuse as well as the muscle cachexia [10]. GSH is a representative endogenous antioxidant, prevents tissue damage by keeping the ROS at low levels at certain cellular concentrations, and is accepted as a protective antioxidant factor in tissues [62]. SOD is one of the antioxidant enzymes that contribute to enzymatic defense mechanisms, and CAT is an enzyme which catalyzes the conversion of H_2_O_2_ to H_2_O [63]. Hence, the inhibition of increased lipid peroxidation and ROS, with increases in GSH content, SOD, and CAT activities in the damaged muscle tissue, is secondarily important for protection against muscle atrophy changes [44, 64, 65]. 4-HNE is an *α*, *β*-unsaturated hydroxyalkenal, which is produced by lipid peroxidation in cells, and it has been used as a valuable tissue lipid peroxidation marker. Also, it is considered as a potential causal agent in numerous diseases, such as chronic inflammation, neurodegenerative diseases, adult respiratory distress syndrome, atherogenesis, diabetes, and different types of cancer [66–68]. Nitrotyrosine is a product of tyrosine nitration mediated by reactive nitrogen species (RNSs) such as peroxynitrite anion and nitrogen dioxide. It is detected in large number of pathological conditions, and it is considered to be a marker of iNOS-dependent RNS-induced nitrative stress [69, 70]. In our study, SF dose-dependently protected NTX-induced gastrocnemius muscle oxidative stresses, the increases in lipid peroxidation and ROS formation, decreases in GSH content, SOD, and CAT activities, and increases in nitrotyrosine and 4-HNE-immunolabeled muscle fibers. Oxymetholone also showed potent antioxidant effects against NTX-induced depletion of antioxidant defense systems, which corresponded well to those of the previous anabolic steroids [71, 72]. Oxidative stress-induced apoptosis of muscle fiber is also involved in the early phase of muscle atrophy regardless of its etiology [11, 12], and caspase-3 and PARP are the key executioners of apoptosis [73, 74]. Therefore, the increase in caspase-3 and PARP immunoreactive muscle fibers in muscle bundles represents their apoptosis and related damage [75, 76]. Therefore, the dose-dependent inhibition of the caspase-3 and PARP immunoreactivities in the NTX gastrocnemius muscle bundles by treatment with EESF as observed in this study is considered as the direct evidence, which indicates that it can preserve the muscle mass through inhibition effects on NTX-induced muscle fiber apoptosis.

The increased production of proinflammatory cytokines, mainly IL-1*β* and TNF-*α*, seems to be involved in inflammatory cachexia and induces the production of prostaglandin E_2_ (PGE_2_), which plays an important role in the inflammatory response. COXs are the key enzymes in regulating the biosynthesis of PGs. There are two COX isoforms, encoded by different genes: COX-1, which is constitutively expressed, and COX-2, which is inducible in response to proinflammatory cytokines and other stimuli. COX-2-induced synthesis of PGs has been associated with chronic inflammation and is also increased in disuse muscle atrophy [77]. Proinflammatory cytokine IL-1*β* exhibits a wide variety of biological actions, including induction of the expression of various growth factors, chemokines, and adhesion molecules [78]. TNF-*α*, a 17-kDa protein, which was first identified as a product of activated macrophages [79], is also one of the well-known proinflammatory cytokines [80, 81]. It is well established that the serum levels of proinflammatory cytokines, particularly IL-1*β* and TNF-*α*, are elevated in cachectic patients contributing to muscle atrophy [4, 8, 9]. In the present study, marked elevations in the gastrocnemius muscle IL-1*β* and TNF-*α* levels with increases in COX-2 and TNF-*α* immunoreactive cells were demonstrated in NTX control mice as compared with sham vehicle control mice, but these elevations in expression levels of proinflammatory cytokines were dose-dependently diminished by treatment with EESF, suggesting that the muscle protective effects of EESF may be mediated by its anti-inflammatory activity, at least partially.

Muscle mass and structure are determined by the balance between protein degradation and synthesis. In the protein degradation pathway, ATP-ubiquitin-dependent proteolysis is the process most probably responsible for muscle wasting [4, 15]. Recent studies have established that muscle-specific E3 ubiquitin ligases such as Atrogin-1 and MuRF1 play critical roles in muscle atrophy [4], and their expression levels are increased in atrophic calf muscles [15, 82] and mice deficient in either Atrogin-1 or MuRF1 are resistant to muscle atrophy [3, 83, 84]. Accordingly, marked elevations in the gastrocnemius muscle Atrogin-1 and MuRF1 mRNA expression levels were demonstrated in NTX control mice as compared with sham vehicle control mice, but these elevations were dose-dependently inhibited by treatment with EESF, which are the direct evidences suggesting that EESF exerts potent muscle protective effects through downregulation of Atrogin-1 and MuRF1, which are involved in muscle protein degradation. Moreover, the insulin-like growth factor 1 (IGF-1)/PI3K/Akt signaling pathway is known to play a pivotal role in activating protein synthesis [4]. PI3K, which is activated by insulin or IGF, in turn activates Akt, a serine/threonine kinase, and its downstream phosphorylates glycogen synthase kinase 3*β* and mammalian target of rapamycin, thereby inducing hypertrophy [8]. In our study, marked downregulation of Akt1 and PI3K mRNA expressions was observed in NTX control mice as denervation-related disuse. However, EESF dose-dependently upregulated the Akt1 and PI3K mRNA expressions as compared with that in NTX control mice, which is the direct evidence suggesting that EESF can activate muscle protein synthesis and confer resistance to NTX-induced disuse muscle atrophy, similar to the effects of oxymetholone.

On the other hand, adenosine is known to modulate various physiological functions of the cardiovascular system [85, 86] and of most tissues including skeletal muscle [87]. It has been proposed that adenosine is involved in both the regulation of blood flow to skeletal muscle [88] and in the synergistic effect of contraction and insulin stimulated glucose uptake in skeletal muscle [89]. The majority of the physiological effects of adenosine are believed to be mediated* via* specific adenosine receptors [90]. Among them, adenosine A1R has been shown to have cytoprotective effects on skeletal muscles [91]. TRPV4 is a member of the TRP channel superfamily [92, 93]. TRPV4 is known to be a Ca^2+^-permeable nonselective cation channel, and it appears to play a mechanosensory or osmosensory role in several musculoskeletal tissues and prevents muscle atrophy or bone loss [93, 94]. NTX significantly decreased the adenosine A1R and TRPV4 mRNA expression levels in the gastrocnemius muscle, also as denervation-related disuse proteolysis; however, EESF upregulated the adenosine A1R and TRPV4 mRNA expression levels in a dose-dependent manner as compared with those in NTX control mice, which can be considered as direct evidences suggesting that it can increase the muscle growth and confer resistance to NTX-induced disuse muscle atrophy, similar to the effects of oxymetholone.

Furthermore, myostatin, a secreted growth differentiation factor, is a member of the TGF-*β* protein family that inhibits muscle differentiation and growth during the process known as myogenesis. Myostatin, a potent negative regulator of muscle growth [15], is produced primarily in skeletal muscle cells, circulates in the blood, and acts on the muscle tissue, by binding to a cell-bound receptor called the activin type II receptor [95, 96]. The sirtuin family of proteins possesses NAD^+^-dependent deacetylase activity and/or ADP ribosyltransferase activity. The seven mammalian sirtuins, SIRT1-7, are localized differentially within the cell and have a variety of functions [97]. Among them, SIRT1 is the most extensively studied member of the family and regulates diverse biological processes ranging from cell proliferation, differentiation, apoptosis, and metabolism [98]. It controls the transcription of the peroxisome proliferator-activated receptor-*γ* coactivator 1 *α* in the skeletal muscle [99] and induces cachexia through inhibiting muscle regeneration [100]. In disuse muscle atrophy, the expressions of myostatin and SIRT1 mRNA have been detected along with decreases in muscle mass [15, 82] and also after NTX in this study. However, these elevations in myostatin and SIRT1 mRNA expression levels were dose-dependently inhibited by treatment with EESF, which can be considered as the direct evidence suggesting that it exerts potent muscle protective effects through downregulation of myostatin and SIRT1.

## 5. Conclusion

The present results suggested that EESF exerts favorable ameliorating effects on muscle atrophy induced by NTX, through anti-inflammatory and antioxidant effects related to muscle fiber protective effects and* via* an increase in protein synthesis and a decrease in protein degradation. These effects of EESF may help improve various muscle atrophies due to various etiologies. The administration of 500 mg/kg EESF led to quite similar favorable muscle preserving effects on NTX-induced muscle atrophy to those of 50 mg/kg oxymetholone, a 17*α*-alkylated anabolic-androgenic steroid, which has been used for treating various muscular disorders, but EESF showed more favorable antioxidant effects in this experiment.

## Figures and Tables

**Table 1:** Oligonucleotides for quantitative RT-PCR used in this study.

Target	5′-3′	Sequence	Size (bp)	Gene ID
Atrogin-1	Forward	CAGCTTCGTGAGCGACCTC	244	67731
	Reverse	GGCAGTCGAGAAGTCCAGTC		

MuRF 1	Forward	GACAGTCGCATTTCAAAGCA	194	433766
	Reverse	GCCTAGCACTGACCTGGAAG		

PI3K p85*α*	Forward	GCCAGTGGTCATTTGTGTTG	236	18708
	Reverse	ACACAACCAGGGAAGTCCAG		

Akt 1	Forward	ATGAACGACGTAGCCATTGTG	116	11651
	Reverse	TTGTAGCCAATAAAGGTGCCAT		

Adenosine A1R	Forward	TGTTCCCAGGGCCTTTCAC	155	11539
	Reverse	TAATGGACTGAGACTAGCTTGACTGGTA		

TRPV4	Forward	CAGGACCTCTGGAAGAGTGC	165	63873
	Reverse	AAGAGCTAGCCTGGACACCA		

Myostatin	Forward	CCTCCACTCCGGGAACTGA	185	17700
	Reverse	AAGAGCCATCACTGCTGTCATC		

SIRT1	Forward	TTCACATTGCATGTGTGTGG	175	93759
	Reverse	TGAGGCCCAGTGCTCTAACT		

18s ribosomal RNA	Forward	AGCCTGAGAAACGGCTACC	252	19791
	Reverse	TCCCAAGATCCAACTACGAG		

**Table 2:** Primary antisera and detection kits used in this study.

Antisera or detection kits	Code	Source	Dilution
Primary antisera			
Anticleaved caspase-3 (Asp175) polyclonal antibody	9661	Cell Signaling Technology Inc., Danvers, MA, USA	1 : 400
Anticleaved PARP (Asp214) specific antibody	9545	Cell Signaling Technology Inc., Danvers, MA, USA	1 : 100
Anti-4-hydroxynonenal polyclonal antibody	Ab46545	Abcam, Cambridge, UK	1 : 100
Antinitrotyrosine polyclonal antibody	06-284	Millipore Corporation, Billerica, CA, USA	1 : 200
Anti-TNF-*α* (4E1) antibody	sc-130349	Santa Cruz Biotechnology, Burlingame, CA, USA	1 : 200
Anti-COX-2 (murine) polyclonal antibody	160126	Cayman Chemical., Ann Arbor, MI, USA	1 : 200
Anti-iNOS (N-20) polyclonal antibody	sc-651	Santa Cruz Biotechnology, Burlingame, CA, USA	1 : 100
Anti-GDF8/myostatin antibody	Ab71808	Abcam, Cambridge, UK	1 : 50
Detection kits			
Vectastain Elite ABC Kit	PK-6200	Vector Lab. Inc., Burlingame, CA, USA	1 : 50
Peroxidase substrate kit	SK-4100	Vector Lab. Inc., Burlingame, CA, USA	1 : 50

**Table 3:** Changes in body weight gains in mice with NTX-induced muscle atrophy.

Groups	Body weights (g)			Body weight gains during the test article treatment period (g) [*B* − *A*]
	At NTX	At 14 days after NTX, first administration [*A*]	Sacrifice [*B*]	
Controls				
Sham	34.48 ± 1.12	32.80 ± 1.26	34.84 ± 1.27	2.04 ± 1.10
NTX	34.33 ± 0.98	32.00 ± 1.18	33.69 ± 1.62	1.69 ± 1.32
Reference				
Oxymetholone	34.71 ± 1.42	32.47 ± 2.31	34.58 ± 3.22	2.11 ± 1.32
EESF treated				
500 mg/kg	34.77 ± 1.59	32.82 ± 2.72	34.53 ± 2.75	1.71 ± 0.98
250 mg/kg	34.26 ± 1.41	32.65 ± 1.89	36.00 ± 3.31^c^	3.35 ± 1.89^ab^
125 mg/kg	34.47 ± 1.68	32.63 ± 2.03	34.40 ± 2.48	1.77 ± 1.19

Values are expressed as mean ± S.D. of 10 mice.

^a^ *p* < 0.05 as compared with sham control mice by the LSD test.

^b^ *p* < 0.01 and ^c^ *p* < 0.05 as compared with NTX control mice by the LSD test.

**Table 4:** Changes in the calf thickness in mice with NTX-induced muscle atrophy.

Groups	Calf thicknesses (mm)			Changes after the test article treatment period (mm) [*B* − *A*]
	At NTX	At 14 days after NTX, first administration [*A*]	Sacrifice [*B*]	
Controls				
Sham	2.70 ± 0.07	2.85 ± 0.10	3.25 ± 0.12	0.40 ± 0.12
NTX	2.69 ± 0.09	2.22 ± 0.06^c^	2.03 ± 0.11^a^	−0.19 ± 0.11^a^
Reference				
Oxymetholone	2.70 ± 0.08	2.22 ± 0.07^c^	2.45 ± 0.10^ab^	0.23 ± 0.11^ab^
EESF treated				
500 mg/kg	2.69 ± 0.05	2.23 ± 0.04^c^	2.44 ± 0.12^ab^	0.21 ± 0.10^ab^
250 mg/kg	2.69 ± 0.07	2.22 ± 0.04^c^	2.33 ± 0.07^ab^	0.11 ± 0.06^ab^
125 mg/kg	2.69 ± 0.06	2.22 ± 0.04^c^	2.30 ± 0.06^ab^	0.08 ± 0.06^ab^

Values are expressed as mean ± S.D. of 10 mice.

^a^ *p* < 0.01 as compared with sham control mice by the LSD test.

^b^ *p* < 0.01 as compared with NTX control mice by the LSD test.

^c^ *p* < 0.01 as compared with sham control mice by the MW test.

**Table 5:** Changes in serum biochemistry in mice with NTX-induced muscle atrophy.

Groups	Serum levels		
	Creatine (mg/dL)	CK (IU/L)	LDH (IU/L)
Controls			
Sham	0.39 ± 0.08	78.10 ± 14.48	484.20 ± 146.42
NTX	0.17 ± 0.05^c^	241.10 ± 42.91^c^	1781.90 ± 184.35^a^
Reference			
Oxymetholone	0.28 ± 0.03^cd^	161.30 ± 37.46^cd^	1086.60 ± 217.18^ab^
EESF treated			
500 mg/kg	0.28 ± 0.04^cd^	162.00 ± 29.06^cd^	1081.90 ± 237.12^ab^
250 mg/kg	0.25 ± 0.04^cd^	181.30 ± 26.23^cd^	1212.60 ± 224.03^ab^
125 mg/kg	0.23 ± 0.04^cd^	195.90 ± 25.67^ce^	1483.80 ± 160.62^ab^

Values are expressed as mean ± S.D. of 10 mice.

^a^ *p* < 0.01 as compared with sham control mice by the LSD test.

^b^ *p* < 0.01 as compared with NTX control mice by the LSD test.

^c^ *p* < 0.01 as compared with sham control mice by the MW test.

^d^ *p* < 0.01 and ^e^ *p* < 0.05 as compared with NTX control mice by the MW test.

**Table 6:** Changes in the gastrocnemius muscle antioxidant defense systems in mice with NTX-induced muscle atrophy.

Groups	Fundus antioxidant defense systems				
	MDA (nM/mg protein)	ROS (RFU/*μ*g protein)	GSH (nM/mg protein)	SOD (nM/mim/mg protein)	CAT (U/mg protein)
Controls					
Sham	1.51 ± 0.41	22.86 ± 5.81	0.67 ± 0.14	24.30 ± 6.15	5.13 ± 1.40
NTX	8.66 ± 1.88^c^	67.11 ± 12.25^a^	0.24 ± 0.07^a^	9.61 ± 1.46^c^	1.45 ± 0.57^c^
Reference					
Oxymetholone	4.11 ± 1.22^cd^	43.08 ± 10.28^ab^	0.45 ± 0.12^ab^	18.13 ± 3.66^cd^	2.63 ± 0.56^cd^
EESF treated					
500 mg/kg	4.38 ± 0.90^cd^	42.35 ± 9.48^ab^	0.49 ± 0.09^ab^	18.11 ± 2.36^cd^	2.66 ± 0.69^cd^
250 mg/kg	5.21 ± 1.17^cd^	49.73 ± 11.92^ab^	0.42 ± 0.08^ab^	15.70 ± 4.05^cd^	2.38 ± 0.62^cd^
125 mg/kg	6.39 ± 0.83^cd^	52.89 ± 11.30^ab^	0.37 ± 0.07^ab^	14.21 ± 1.97^cd^	2.06 ± 0.40^ce^

Values are expressed as mean ± S.D. of 10 mice.

^a^ *p* < 0.01 as compared with sham control mice by the LSD test.

^b^ *p* < 0.01 as compared with NTX control mice by the LSD test.

^c^ *p* < 0.01 as compared with sham control mice by the MW test.

^d^ *p* < 0.01 and ^e^ *p* < 0.05 as compared with NTX control mice by the MW test.

**Table 7:** Changes in the gastrocnemius muscle cytokine content in mice with NTX-induced muscle atrophy.

Groups	Muscle cytokine content (pg/mg protein)	
	IL-1*β*	TNF-*α*
Controls		
Sham	1.64 ± 0.55	75.67 ± 16.06
NTX	5.45 ± 1.51^a^	244.76 ± 43.21^a^
Reference		
Oxymetholone	2.58 ± 0.56^ab^	137.00 ± 34.23^ab^
EESF treated		
500 mg/kg	2.70 ± 0.71^ab^	136.43 ± 27.85^ab^
250 mg/kg	3.27 ± 0.49^ab^	149.86 ± 15.01^ab^
125 mg/kg	3.99 ± 0.50^ab^	174.87 ± 24.71^ab^

Values are expressed as mean ± S.D. of 10 mice.

^a^ *p* < 0.01 as compared with sham control mice by the MW test.

^b^ *p* < 0.01 as compared with NTX control mice by the MW test.

**Table 8:** Changes in the gastrocnemius muscle mRNA expression levels in mice with NTX-induced muscle atrophy.

Targets	Groups					
	Controls		Reference	EESF treated mice (mg/kg)		
	Sham	NTX	Oxymetholone	500	250	125
Atrogin-1	1.05 ± 0.11	4.41 ± 1.06^d^	2.24 ± 0.28^df^	2.29 ± 0.59^df^	2.85 ± 0.61^df^	3.19 ± 0.78^df^
MuRF 1	1.04 ± 0.13	3.81 ± 0.69^a^	2.21 ± 0.43^ac^	2.20 ± 0.55^ac^	2.37 ± 0.42^ac^	2.95 ± 0.61^ac^
PI3K p85*α*	0.95 ± 0.11	0.74 ± 0.12^d^	1.54 ± 0.64^ef^	1.58 ± 0.73^f^	1.05 ± 0.13^f^	0.93 ± 0.12^f^
Akt 1	0.97 ± 0.11	0.60 ± 0.18^a^	0.88 ± 0.14^c^	0.88 ± 0.10^c^	0.80 ± 0.10^ac^	0.78 ± 0.10^ac^
Adenosine A1R	0.98 ± 0.12	0.57 ± 0.10^a^	0.97 ± 0.13^c^	0.98 ± 0.14^c^	0.84 ± 0.15^bc^	0.79 ± 0.11^ac^
TRPV4	0.99 ± 0.09	0.45 ± 0.12^a^	0.77 ± 0.13^ac^	0.77 ± 0.11^ac^	0.69 ± 0.11^ac^	0.64 ± 0.12^ac^
Myostatin	1.00 ± 0.11	7.65 ± 1.53^d^	3.25 ± 0.99^df^	3.20 ± 0.71^df^	4.16 ± 0.78^df^	5.36 ± 0.49^df^
SIRT1	1.03 ± 0.14	6.83 ± 1.07^d^	3.80 ± 0.81^df^	3.83 ± 0.95^df^	4.35 ± 1.10^df^	5.22 ± 0.79^df^

Values are expressed as mean ± S.D. of six mice, relative expressions/18s ribosomal RNA.

^a^ *p* < 0.01 and ^b^ *p* < 0.05 as compared with intact control mice by the LSD test.

^c^ *p* < 0.01 as compared with NTX control mice by the LSD test.

^d^ *p* < 0.01 and ^e^ *p* < 0.05 as compared with intact control mice by the LSD test.

^f^ *p* < 0.01 as compared with NTX control mice by the LSD test.

**Table 9:** Changes in the gastrocnemius muscle histomorphometrical analysis in mice with NTX-induced muscle atrophy.

Groups	Histomorphometry of the gastrocnemius muscle bundles		
	Mean muscle fiber diameter (*μ*m/fiber)	Number of infiltrated inflammatory cells (cells/mm^2^)	Regions occupied by collagen fibers (%/mm^2^)
Controls			
Sham	53.23 ± 7.21	5.90 ± 1.52	5.98 ± 3.06
NTX	14.81 ± 3.14^a^	327.10 ± 96.04^a^	40.11 ± 6.34^a^
Reference			
Oxymetholone	29.64 ± 2.30^ab^	86.60 ± 41.21^ab^	23.04 ± 2.66^ab^
EESF treated			
500 mg/kg	29.94 ± 3.02^ab^	89.00 ± 22.67^ab^	23.50 ± 4.11^ab^
250 mg/kg	29.90 ± 3.74^ab^	124.00 ± 15.61^ab^	27.50 ± 5.30^ab^
125 mg/kg	24.34 ± 4.23^ab^	148.10 ± 31.97^ab^	31.34 ± 6.01^ab^

Values are expressed as mean ± S.D. of 10 mice.

^a^ *p* < 0.01 as compared with sham control mice by the MW test.

^b^ *p* < 0.01 as compared with NTX control mice by the MW test.

**Table 10:** Changes in the gastrocnemius muscle immunohistomorphometrical analysis in mice with NTX-induced muscle atrophy.

Antibody	Groups					
	Controls		Reference	EESF treated mice (mg/kg)		
	Sham	NTX	Oxymetholone	500	250	125
Caspase-3 (fibers/mm^2^)	3.70 ± 1.49	40.70 ± 5.85^c^	19.40 ± 4.09^cd^	19.60 ± 2.50^cd^	23.10 ± 3.90^cd^	31.40 ± 3.75^cd^
PARP (fibers/mm^2^)	6.10 ± 1.60	73.50 ± 11.33^c^	44.60 ± 11.17^cd^	43.70 ± 12.27^cd^	53.90 ± 10.50^cd^	60.80 ± 6.43^cd^
NT (fibers/mm^2^)	8.70 ± 2.00	66.70 ± 14.01^c^	30.60 ± 11.09^cd^	33.50 ± 11.42^cd^	44.90 ± 10.48^cd^	50.86 ± 11.19^ce^
4-HNE (fibers/mm^2^)	5.70 ± 2.16	74.60 ± 12.38^c^	42.90 ± 10.34^cd^	42.50 ± 12.25^cd^	52.40 ± 10.46^cd^	61.30 ± 10.79^ce^
iNOS (fibers/mm^2^)	6.40 ± 2.01	71.90 ± 12.13^a^	36.30 ± 11.47^ab^	35.90 ± 10.35^ab^	45.70 ± 10.53^ab^	57.00 ± 11.81^ab^
COX-2 (cells/mm^2^)	10.80 ± 4.78	259.80 ± 30.90^c^	194.10 ± 30.50^cd^	154.20 ± 25.18^cd^	185.10 ± 20.63^cd^	226.20 ± 25.13^ce^
TNF-*α* (cells/mm^2^)	32.40 ± 13.20	231.20 ± 41.87^a^	171.10 ± 18.39^ab^	148.20 ± 29.95^ab^	183.70 ± 22.07^ab^	191.30 ± 21.54^ab^
Myostatin (fibers/mm^2^)	3.30 ± 1.34	45.30 ± 10.65^c^	17.40 ± 4.27^cd^	18.50 ± 6.95^cd^	27.20 ± 5.12^cd^	32.10 ± 10.92^cd^

Values are expressed as mean ± S.D. of 10 mice.

^a^ *p* < 0.01 as compared with sham control mice by the LSD test.

^b^ *p* < 0.01 as compared with NTX control mice by the LSD test.

^c^ *p* < 0.01 as compared with sham control mice by the MW test.

^d^ *p* < 0.01 and ^e^ *p* < 0.05 as compared with NTX control mice by the MW test.
